# Pharmaceutical Care Increases Time in Therapeutic Range of Patients With Poor Quality of Anticoagulation With Warfarin

**DOI:** 10.3389/fphar.2018.01052

**Published:** 2018-09-21

**Authors:** Leiliane Rodrigues Marcatto, Luciana Sacilotto, Letícia Camargo Tavares, Mirella Facin, Natália Olivetti, Celia Maria Cassaro Strunz, Francisco Carlos Costa Darrieux, Maurício Ibrahim Scanavacca, Jose Eduardo Krieger, Alexandre Costa Pereira, Paulo Caleb Junior Lima Santos

**Affiliations:** ^1^Laboratory of Genetics and Molecular Cardiology, Faculdade de Medicina FMUSP, Heart Institute (InCor), Universidade de São Paulo, São Paulo, Brazil; ^2^Arrythmia Unit, Faculdade de Medicina FMUSP, Heart Institute (InCor), Universidade de São Paulo, São Paulo, Brazil; ^3^Clinical Laboratory, Heart Institute (InCor), Faculdade de Medicina FMUSP, Heart Institute (InCor), Universidade de São Paulo, São Paulo, Brazil; ^4^Department of Pharmacology, Universidade Federal de São Paulo – Escola Paulista de Medicina, São Paulo, Brazil

**Keywords:** pharmaceutical care, warfarin, poor quality of anticoagulation, time in the therapeutic range, pharmacist management

## Abstract

Thromboembolic events are associated with high mortality and morbidity indexes. In this context, warfarin is the most widely prescribed oral anticoagulant agent for preventing and treating these events. This medication has a narrow therapeutic range and, consequently, patients usually have difficulty in achieving and maintaining stable target therapeutics. Some studies on the literature about oral anticoagulant management showed that pharmacists could improve the efficiency of anticoagulant therapy. However, the majority of these studies included general patients retrospectively. The aim of this study was to prospectively evaluate a pharmacist’s warfarin management in patients with poor quality of anticoagulation therapy (Time in the Therapeutic Range- TTR < 50%). We included 268 patients with atrial fibrillation (AF) and without stable dose of warfarin (TTR < 50%, based on the last three values of International Normalized Ratio-INR). We followed them up for 12 weeks, INR values were evaluated and, when necessary, the dose adjustments were performed. During the first four visits, patient’s INR was measured every 7 days. Then, if INR was within the target therapeutic range (INR: 2–3), the patient was asked to return in 30 days. However, if INR was out the therapeutic target, the patient was asked to return in 7 days. Adherence evaluation was measured through questionnaires and by counting the pills taken. Comparison between basal TTR (which was calculated based on the three last INR values before prospective phase) and TTR of 4 weeks (calculated by considering the INR tests from visits 0 to 4, in the prospective phase of the study) and basal TTR and TTR of 12 weeks (calculated based on the INR tests from visits 0 to 12, in the prospective phase of the study) revealed significant statistical differences (0.144 ± 0.010 vs. 0.382 ± 0.016; and 0.144 ± 0.010 vs. 0.543 ± 0.014, *p* < 0.001, respectively). We also observed that the mean TTR of 1 year before (retrospective phase) was lower than TTR value after 12 weeks of pharmacist-driven treatment (prospective phase) (0.320 ± 0.015; 0.540 ± 0.015, *p* < 0.001). In conclusion, pharmaceutical care was able to improve TTR values in patients with AF and poor quality of anticoagulation with warfarin.

## Introduction

Thromboembolic events are associated with more than one-half million hospitalizations in the United States each year, and are a contributing cause in 100,000 or more deaths (National Heart, Lung, and Blood Institute, 2008). Increased risk of these events may be caused by obesity, genetic factors, medications, hospitalization and comorbidities such as AF (The Lancet Haematology, 2015; Lutsey et al., 2018).

Atrial fibrillation (AF), the most common cardiac arrhythmia in clinical practice, and is also associated with high mortality and morbidity index because it commonly leads to thromboembolic events and heart failure. AF is frequently associated with palpitations and fluttering symptoms, although AF remains asymptomatic for many patients. In 2010, it was estimated that there are 33.5 million people in the world with AF (Feinberg et al., 1995; Chugh et al., 2014; Shantsila and Lip, 2016). In addition, patients older than 80 years have 5–15% increased prevalence of AF (Camm et al., 2010).

Preventing thromboembolic events in patients with AF is crucial. In fact, Lutsey et al. did a bidirectional relationship between AF and venous thromboembolism (VTE) and showed that AF was associated with greater risk of subsequent incident VTE (hazard ratio [95% CI], 1.71 [1.32–2.22]), in a way that the association was stronger in African descendents and during the first 6 months after AF diagnosis (2.30 [1.48–3.58]; 5.08 [3.08–8.38]). Furthermore, in the presence of AF, the incidence rate of VTE is 6.3 per 1,000 persons-year. By contrast, without AF the incidence rate of VTE is 2.4 per 1,000 persons-year (Lutsey et al., 2018). Another study compared the risk of ischemic stroke and intracranial hemorrhage for AF patients aged ≥90 years and patients without AF. Results showed that AF patients had an increased risk of ischemic stroke compared to non-AF patients (event number/patient number; incidence = 742/11,064; 5.75%/year vs. 1,399/14,658; 3.00%/year; hazard ratio [HR] 1.93, 95% confidence interval [CI] 1.74–2.14) (Chao et al., 2018).

In order to prevent thromboembolic events in AF patients, clinicians usually adopt the anticoagulation therapy, which is effective in reducing the risk of thromboembolic events (Wolf et al., 1991). Anticoagulation therapy with warfarin, a vitamin K antagonist, remains widely used by patients with AF (Ageno et al., 2012; Kirchhof et al., 2016). A published meta-analysis showed that warfarin was able to reduce stroke events by approximately 60% in patients with AF (Hart et al., 2007). Nevertheless, this pharmacotherapy presents a narrow therapeutic index, extensive drug and food interactions and large inter-individual variability in dose requirements, with intensive monitoring necessary to be effective and avoid adverse effects (Wells et al., 1994; Wan et al., 2008; Guyatt et al., 2012; Soares et al., 2012; Santos et al., 2013; Marcatto et al., 2016b; Tavares et al., 2018). In this sense, a study from Lip et al investigated the incidence and risk of adverse events in a real world setting in AF patients using warfarin. The authors showed that a real world cohort had higher risk of adverse events than clinical trial cohorts (hazard ratio [HR], 6.32; 95% CI, 2.84–14.03 for major bleeding; HR, 3.56, 95% CI, 1.22–10.42 for ischemic stroke; HR, 5.13, 95% CI, 3.02–8.69 for all-cause mortality) (Rivera-Caravaca, 2018). Thus, because of the high risk of thromboembolic events in AF patients, guidelines recommend that clinicians use the international normalized ratio (INR) test for regularly monitoring the effectiveness of anticoagulation treatment with warfarin, as INR values outside of the target therapeutic range increase the risk of both bleeding and thromboembolic events (Ageno et al., 2012).

A commonly utilized method for evaluating the quality of treatment with warfarin is the percentage of time in the therapeutic range (TTR) by the Rosendaal’s method (Rosendaal et al., 1993). Studies have shown that higher TTR is associated with lower incidence of warfarin-related complications, such as hemorrhagic and thromboembolic events (White et al., 2007).

In this scenario, several studies are currently evaluating pharmacist-based practices that may improve the quality of warfarin treatment (Lee et al., 2016; Aidit et al., 2017). For example, Aidit and colleagues evaluated the pharmacist-led warfarin management over the standardized treatment protocol. They showed that there is a significant positive association between pharmacist-driven management and stable TTR and INR parameters (Aidit et al., 2017). Furthermore, a review of the efficacy and safety of the pharmacist-led management, including 26 studies, found evidence that pharmacist-driven management leads to better outcomes when compared to usual care (Lee et al., 2016). Another recent study showed that increased TTR was associated with lower adverse events due to pharmacist-driven management (Phelps et al., 2018).

Although studies have shown that pharmaceutical care driving the anticoagulation therapy with warfarin is beneficial, the majorities of them are retrospective and include general patients. To our knowledge, prospective studies with patients with poor quality of treatment with warfarin were never studied before, and they are the group with the highest risk of adverse events. Therefore, the aim of the study was to evaluate a pharmacist’s warfarin management in patients with poor quality of anticoagulation therapy.

## Materials and Methods

This study included a cohort of 268 patients with low TTR (<50%) from the Heart Institute (InCor), Faculdade de Medicina FMUSP, Universidade de São Paulo. The study protocol was approved by the Ethics Committee for Medical Research on Human Beings of the Heart Institute (InCor), Faculdade de Medicina FMUSP, Universidade de São Paulo (SDC 4033/14/013). Signed informed consents were obtained from all participants.

We included patients with AF, over 18 years, and with TTR < 50%, based on the last three values of INR. Patients were excluded if they presented liver and/or kidney dysfunctions, alcoholism, use of another anticoagulant, and/or chemotherapy treatment. Furthermore, if the patient administered amiodarone, a dosage change within 1 week of entering the study was considered an exclusion criterion.

This study consists of: (i) a retrospective descriptive study, involving the same patients selected for the prospective study, 1 year before they started the follow-up with the pharmacist, and, (ii) a prospective descriptive study of a 12-week, pharmacist-driven warfarin therapy.

### Retrospective Phase

Time in the therapeutic range was evaluated for a period of 1 year before the start of the prospective phase. For calculating the patient’s retrospective TTR, medical records were used for collecting the INR values. At the retrospective phase, patients receiving warfarin therapy were routinely treated and managed by physicians. Medical residents provided drug information before patients visited the physician. Physicians performed warfarin dosing adjustment, adverse event examination, and follow-up appointment. After that, patients went to the pharmacy department to receive drugs with general instruction on how to use warfarin. Clinical pharmacists were not involved in this process. Therefore, we utilized TTR value of 1 year before the implementation of pharmacist’s warfarin management as a control TTR, for comparing them with the TTR after the implementation of pharmacist’s warfarin management.

### Prospective Phase

**FIGURE 1:**
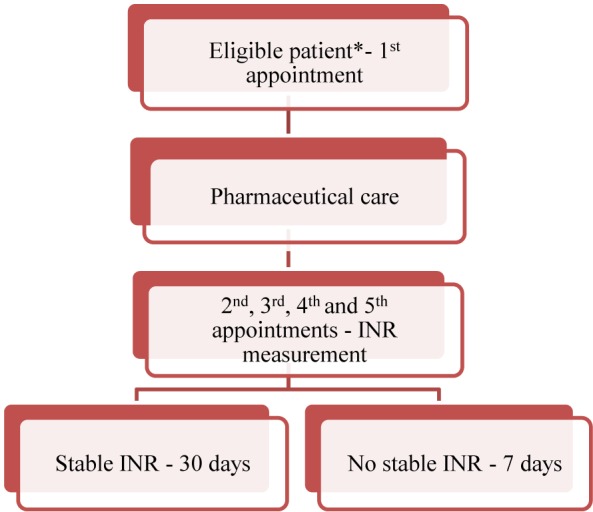
Protocol’s study design. **^∗^**The patients were included in the study if they met the inclusion criteria. Pharmaceutical care consisted of providing the medication, explaining about warfarin administration requirements and the AF disease, as well as informing the individual’s dosage by phone on the day following the first appointment. On the 1st, 2nd, 3rd, and 4th visit, which occurred at an interval of 7 days each, INR was measured. If the INR was stable, the patient returned in 30 days. But, if the INR was not stable, the patient returned in 7 days. The follow-up of each individual lasted for 12 weeks.

**Figure 1** shows the study design of prospective phase. A clinical pharmacist followed up patients with TTR < 50% for 12 weeks. During this study period, for every appointment with a cardiologist, the patients also received pharmacist’s intervention through one-on-one visit. In this phase, clinical pharmacists: assessed and reported all drug–drug interaction, drug–food interaction and medication mistakes that could lead to preventable events; evaluated the adherence and adverse events and fixed non-adherence and adverse event issues; provided patient education with the orientation about how using warfarin correctly; and performed warfarin dosing adjustment according to established guidelines (Ansell et al., 2008; Cushman et al., 2011). The clinical pharmacist also used the EP mobile tool for assisting in the management of the weekly dose (Horton and Bushwick, 1999). Patients with INR values <1.8 or >3.2 underwent dose adjustment. For patients with an INR value between 1.8 and 2.0 or 3.0 and 3.2, warfarin dose was maintained and INR tests were continued every 7 days. Then, if the patient continued to demonstrate values between 1.8 and 2.0 or 3.0 and 3.2, warfarin dose was changed. At each patient appointment, the pharmacist checked the drug adherence by counting pills and adverse events using patient self-reporting information. The average weekly warfarin dose was changed according to patient’s INR value: ≤1.5, increased by 20%; >1.5 to < 2.0, increased by 5%; >3.0 to 3.5, decreased by 5%; >3.5 to <6.0, withheld one dose, decreased by 15%; ≥6.0, according to guidelines (Ansell et al., 2008; Cushman et al., 2011). In addition, the clinical pharmacist recommended alternative drugs if it was necessary, although the cardiologists were who made the final decision about the treatment.

At visit 0, the cardiologist selected the eligible patient according to the inclusion criteria. Then, the pharmacist re-checked if he/she met the inclusion criteria and explained the study to the patient. If the patient agreed to participate in the study, the informed consent was signed. The clinical pharmacist applied questionnaires, which included variables such as comorbidities, previous adverse events, quantity of vitamin K intake, medications and others. Creatinine and liver transaminases were measured for confirming that the patient had not liver and/or renal dysfunction at a clinical laboratory in the Heart Institute. Then, the clinical pharmacist dispensed the medication (warfarin) to the patient, provided instructions about how to correctly administer it, evaluated possible drug interactions, evaluated possible drug–food interactions, performed dose adjustment, according to guidelines and requested a new INR measurement in 7 days. At the visit 1, 2, 3, and 4, which occurred each 7 days, a new INR test was made and warfarin dosage was adjusted, if it was necessary. In addition, the clinical pharmacist evaluated possible adverse events and adherence. After visit 4, if the patient’s INR was within the target therapeutic range (INR: 2–3), the next INR measurement was assessed after 30 days. If the patient’s INR was not within the target therapeutic range, the warfarin dose adjustment was performed and the INR measurement was repeated after 7 days, until 12 weeks of follow-up were completed (Marcatto et al., 2016a).

Oral anticoagulant therapy was assessed by the prothrombin time (PT) through and automated coagulometer at the clinical laboratory of the Heart Institute. Venous blood samples were collected in tubes containing sodium citrate 3.8%. INR calculation was obtained by the ratio of a patient’s prothrombin time to a normal (control) sample, raised to the power of the international sensitivity index (ISI) value for the analytical system being used. Past INR values were checked on electronic medical records. TTR was calculated by the Rosendaal’s method, which uses linear interpolation to assign an INR value to each day between successive observed INR values (Rosendaal et al., 1993).

Each patient had an individual medical record. This record provided information on current warfarin dose (mg/week), daily dose, and INR values. In addition, each patient had a personal card to register the daily warfarin dose. The information gathered in the study was managed using REDCap (Research Electronic Data Capture) tools hosted at Heart Institute (InCor), Faculdade de Medicina FMUSP, Universidade de São Paulo. REDCap is a secure, web-based application designed to support data capture for research studies (Harris et al., 2009).

### Outcomes

The outcomes were the percentages of TTR in patients with AF at different time points of the follow-up. We evaluated the basal TTR (based on the last three INR exams taken before the patient entering in the prospective phase of the study, with a cut-off for selection of TTR < 50%); the TTR of 4 weeks after the initiation of the protocol; TTR from 4 to 12 weeks; and TTR of 12 weeks. We also calculated the percentage of TTR in the same patients 1 year before they started the follow-up with the pharmacist (retrospective phase).

### Statistical Analysis

Descriptive analyses were used to present the characteristics of the patients. Data are presented as mean and standard deviation (SD) or standard error (SE) of the mean. Kolmogorov Smirnov and Shapiro-Wilk tests were used to assess normality. The TTR variable did not present a normal distribution. For this reason, non-parametric tests were performed. For comparison of mean TTR values, a Wilcoxon signed-rank test was performed, by comparing two by two. Statistical analysis was carried out using SPSS software (v. 16.0, IBM, New York, NY, United States). The level of significance was set at *p* ≤ 0.05.

## Results

**Table 1** summarizes the demographic and clinical characteristics of the 268 patients enrolled in the study. The mean age was 66.6 ± 0.7 years; 47.6% were female. The predominant self-reported race was white (48.7%), followed by intermediate (brown) (38.6%) and black (11.2%).

**Table 1:** Demographic and clinical characteristics of the patients (*n* = 268).

Variables	Values
Female (%)	47.6
**RACE (%)**	
White	48.7
Intermediate (Brown)	38.6
Black	11.2
Others	1.5
Amiodarone use (%)	20.2
Inductor use (%)	13.9
Current smoker (%)	9.0
Alcohol intake (%)	13.9
Vitamin K intake (%)	37.5
Diabetes (%)	31.5
Hypertension (%)	81.6
Dyslipidemia (%)	56.2
Age (years)	66.6 ± 0.7
Weight (kg)	78.2 ± 1.0
Height (cm)	165 ± 0.6

*Data are presented as % or mean ± SD.*

**FIGURE 2:**
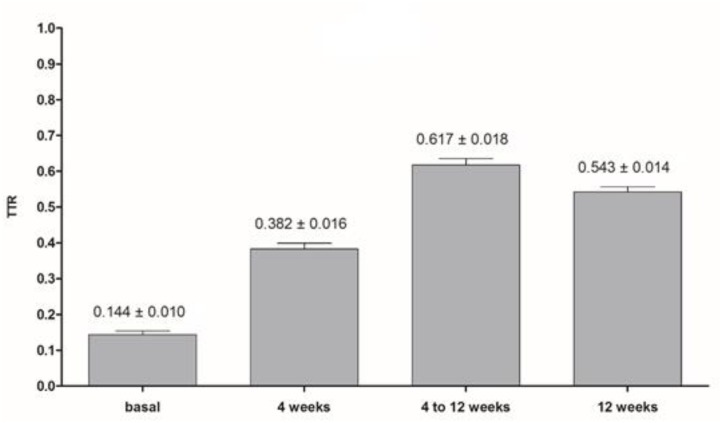
Comparison between the basal and the TTRs calculated in different times (TTR 4 weeks: based on INR tests from visits 0 to 4; TTR 4–12 weeks: based on INR tests from visits 4–12; TTR 12 weeks: based on INR tests from visits 0–12). Wilcoxon signed-rank test was performed for comparison two by two (basal and TTR 4 weeks; basal and TTR 4–12 weeks; and, basal and TTR 12 weeks); all *p*-values were *p* < 0.001. Data are expressed as mean ± SE. Basal TTR is the TTR calculated with the 3 last INR tests before entering at prospective phase of the study with a cut-off for selection TTR < 0.500, TTR 4 weeks (TTR calculated with the INR tests from visits 0 to 4 at prospective phase of the study), TTR 4–12 weeks (TTR calculated with the INR tests from visits 4 to 12 at prospective phase of the study) and TTR 12 (TTR calculated with the INR tests from visits 0 to 12 at prospective phase of the study).

**FIGURE 3:**
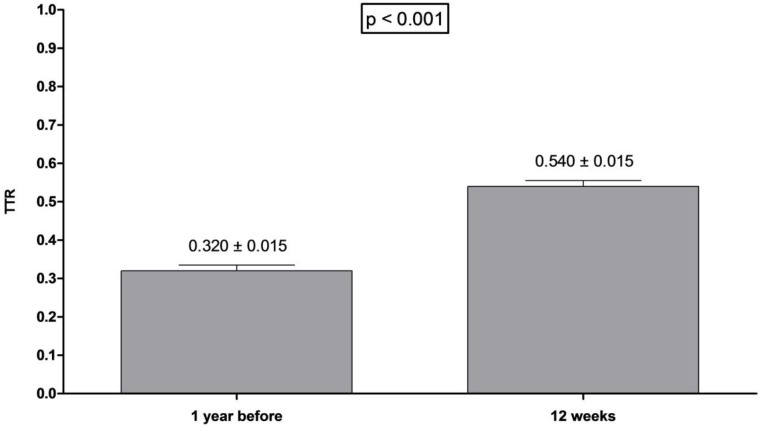
Comparison between TTR of 1 year before and 12 weeks in patients included at the protocol. Wilcoxon signed-rank test was performed. Data are expressed as mean ± SE. TTR of 1 year before was calculated for the period of 1 year prior to the patient entering the protocol. TTR of 12 weeks was calculated 12 weeks after protocol start.

**Figure 2** shows a comparison between TTRs that were calculated in different times. The basal TTR (TTR calculated based on the 3 last INR tests before the prospective phase of the study), TTR 4 weeks (TTR calculated with the INR tests from visits 0 to 4 in the prospective phase of the study), TTR 4–12 weeks (TTR calculated based on the INR tests from visits 4 to 12 in the prospective phase of the study) and TTR 12 (TTR calculated by using the INR tests from visits 0 to 12 in the prospective phase of the study). We observed that the mean of TTR 12 weeks and the mean of TTR 4 weeks were significantly higher than the basal TTR mean (54.3% vs. 14.4%; *p* < 0.001; 38.2% vs. 14.4%; *p* < 0.001, respectively), suggesting that the pharmacist-driven management improved warfarin therapy. Moreover, for assuring that the pharmacist-driven management really improved warfarin therapy, we used as a measure control the TTR calculated for 1 year before the prospective phase has started (retrospective phase). **Figure 3** shows that the TTR after 12 weeks of pharmacist care was significantly higher than the TTR of 1 year before (54.3% vs. 32.0%; *p* < 0.001) (**Figure 3**).

We also observed that the medians of the basal TTR and the TTR of 1 year before are lower than the TTR in the prospective phase. Furthermore, the mean TTR after the prospective phase is higher (**Supplementary Figure 1**).

In addition, we compared the antepenultimate, penultimate and last INR before the prospective phase of the study, as well as the INR at 4 and 12 weeks after protocol start. Boxplots reveal that the antepenultimate INR presents larger distribution of data than the others INR measurements, which suggests that the patients presented an increased risk for thromboembolic events and bleeding events before pharmacist-driven management. Furthermore, the median of the three INR measurements before pharmacist-driven management was lower than the INR at 4 and 12 weeks after protocol start (**Supplementary Figure 2**), indicating that the patients presented an increased risk for thromboembolic events.

## Discussion

Our findings demonstrated that implementation of pharmaceutical warfarin therapy management for patients with poor quality of anticoagulation was able to improve TTR values when compared to the TTR of the same patient 1 year before starting the follow-up with the pharmacist. The major differences of our study with previous that have already shown a significant improvement in coagulation therapy are: our study is composed of two phases (retrospective and prospective phases), rather than only retrospective analysis; these previous studies included general patients, whereas our study included only patients with unstable dose and poor quality of warfarin therapy (TTR < 50%); and they are randomized trials, whereas our study was performed by using paired comparisons (the subjects’ TTR were assessed and compared before and after pharmacist-driven warfarin therapy management, in a way that before receiving pharmacist-guidance the patients received warfarin traditional treatment by physicians) (Saokaew et al., 2012; Verret et al., 2012; Katada et al., 2017).

In the current study, after 12 weeks of a pharmacist follow-up the TTR mean of the patients receiving warfarin increased from 14.4 to 54.3%. In spite of the fact that we observed a significant TTR improvement at the end of the protocol, the final TTR did not reach the adequate level recommended for warfarin therapy (between 58 and 65% and an optimal control of >70% TTR) (Connolly et al., 2008). The final TTR demonstrated in our study was not high enough, probably because we have only selected patients with very low TTR, who can have peculiarities (intrinsic and extrinsic factors) that make it difficult to achieve optimal quality. In addition, an interesting meta-analysis suggests that for capturing all effects of pharmacist-driven management, a follow-up time of ≥6 months is necessary (Saokaew et al., 2010). Here, we followed-up for 12 weeks. Despite this, the TTR of the period between the 4th and 12th weeks of protocol was 61.7%, which is adequate according to the recommended level.

In fact, Connolly’s study showed that in developing countries the TTR tends to be lower compared with developed countries. Specifically, the authors demonstrated that the TTR mean in Brazil was 47.1% (Connolly et al., 2008), which is even numerically lower than the final TTR reached in the present study. Nevertheless, our study selected a group of patients with poor quality that does not reflect the selection location (which has optimal TTR mean) and Brazilian patients in general.

TTR improvement reached after implementation of pharmaceutical care for patients with poor warfarin therapy quality is very important, as studies have demonstrated that patients with low TTR present higher risks for thromboembolic and bleeding events (Pokorney et al., 2015). Furthermore, another study showed that patients with TTR < 45% presented increased rates of thromboembolic and major bleeding events when compared with patients with TTR > 65% (RR 2.8, CI 1.9–4.3, *P* < 0.001) (Veeger et al., 2005).

Additionally, a meta-analysis showed that a 7% improvement in TTR would lead to a reduction of 1 major bleeding event per 100 patient-years, and a 12% improvement in TTR would lead to a reduction of 1 thromboembolic event per 100 patient-years (Wan et al., 2008). Thus, we can say that, in this study, the pharmaceutical care was able to decrease the risk of major bleeding and thromboembolic events, as after 4 weeks of pharmacist-guided treatment the TTR increased by 23.8%, and after 12 weeks of follow-up, we observed a TTR improvement of 39.9%.

The TTR improvement of 39.9% reported in the present study after 12 weeks of pharmacist follow-up is more promising than that reported in other similar studies. For example, Víquez-Jaikel and colleagues demonstrated an increase of 29.5% in TTR mean after implementation of pharmacist-driven management when compared to usual care (Viquez-Jaikel et al., 2016). Furthermore, Motiycka and contributors showed a TTR improvement of 10%, and Young et al. (2011) reported an increase of 8% in TTR mean (Motycka et al., 2011). Nevertheless, these studies compared groups of treatment (pharmacist-driven vs. usual care) and considered different inclusion criteria than those considered by us. In contrast, in our study we compared the same patients, at different time points of the pharmacist follow-up. Thus, the possible reasons for the significant TTR improvement observed in our study may be due to the good interaction between the patients and the pharmacist, the adhesion of the patient to the treatment that was checked during the entire follow-up and the pharmacist-driven educational support about the disease and treatment that was given to the patients.

Recent studies have shown that the pharmacist-driven management of warfarin therapy is beneficial, in agreement with the key finding of this study. Downing et al. (2016) assessed the effect of the implementation of pharmacist-driven warfarin dosing protocol in a retrospective study and they showed that the implementation was able to increase TTR and decrease the time to achieve the target therapeutic range. A prospective study evaluated the impact of the clinical pharmacist and physician on improving patient’s education and achieving the therapeutic INR level. The authors showed that therapeutic INR and general drug knowledge scores were significantly higher after clinical pharmacist and physician counseling (Choumane et al., 2018). In addition, a Brazilian study showed that the patients achieved an adequate TTR after the pharmacist-driven management (64.3%) (de Lima Silva et al., 2017).

Our study has some limitations. First, the ideal design of an interventional study is the randomized control trial. However, our intention was to compare the patients against their own data at different time points: without pharmacist-driven management and with pharmacist-driven management, in order to observe whether these patients with poor quality of treatment could improve their anticoagulant therapy after pharmacist care. Before the follow up with clinical pharmacist, the group of patients with poor quality of treatment received traditional treatment. TTR values of this patient group 1 year before the follow-up with the clinical pharmacist indicated poor quality of treatment. Moreover, selecting a group of patients with poor anticoagulation therapy quality, as a control group, and not supporting them with the best clinical practice would not be ethical. Second, articles in the literature use general warfarin patients to compose their cohorts and our cohort are composed of patients with poor anticoagulation, who need more attention in the management. Therefore, our findings could not be similar for new-diagnosed and general patients.

## Conclusion

In conclusion, the implementation of pharmaceutical care for patients with AF and poor quality anticoagulation with warfarin increases TTR and, probably reduces the risk of adverse events. This benefit is important for Brazilian health policy and justifies the practice of pharmacist-driven management.

## Author Contributions

All authors have contributed substantially to the conception and design of this paper. LM attended to all the patients, collected and analyzed the data, and wrote the paper. LS, MF, NO, and FD recruited the patients, collected data, and critically revised the manuscript. CS, MS, JE, and AC provided the facilities. PC and LT analyzed the data and critically revised the manuscript.

## Conflict of Interest Statement

The authors declare that the research was conducted in the absence of any commercial or financial relationships that could be construed as a potential conflict of interest.
