# Supplementary material for: Pharmaceutical Care Increases Time in Therapeutic Range of Patients With Poor Quality of Anticoagulation With Warfarin
Source: Front Pharmacol. 2018 Sep 21;9:1052. doi: 10.3389/fphar.2018.01052 (PMC6160801; doi:10.3389/fphar.2018.01052)
Supplement: Supplementary file 1 [file Data_Sheet_1.DOCX]

Supplementary Material

**Pharmaceutical care increases time in therapeutic range of patients with poor quality of anticoagulation with warfarin**

**Leiliane Rodrigues Marcatto, Luciana Sacilotto, Letícia Camargo Tavares, Mirella Facin, Natália Olivietti, Celia Maria Cassaro Strunz, Francisco Carlos Costa Darrieux, Maurício Ibrahim Scanavacca, Jose Eduardo Krieger, Alexandre Costa Pereira, Paulo Caleb Junior Lima Santos^*^**

**^*^ Correspondence:**

Dr. Paulo Caleb Junior Lima Santos

paulo.caleb@unifesp.br

**1 SUPPLEMENTARY FIGURES AND TABLES**


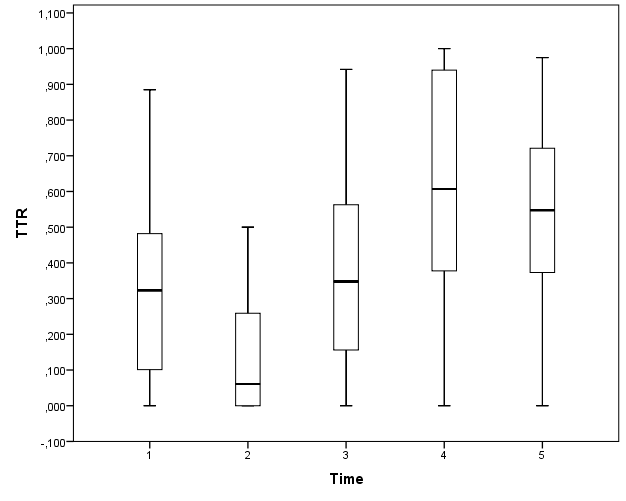


**Supplementary figure 1.** **Comparison between the TTR at 1 year before (1), basal TTR (2), TTR at 4 weeks (3), TTR between 4 and 12 weeks (4), TTR at 12 weeks**

**(5).** Basal TTR is the TTR that the patient presented prior to entering the study, considering the three last INR values (cut-off for selection was TTR<0.500). TTR of 4 weeks, TTR of 4 to 12 weeks, and TTR of 12 weeks after protocol start were calculated.


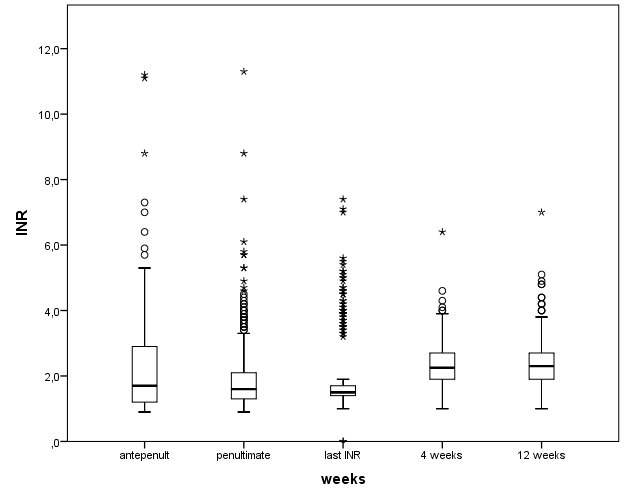


**Supplementary figure 2.** **Comparison between the mensurations of INR at different times.** Antepenultimate, penultimate and last INR is the INR measured before the protocol start and INR at 4 weeks and INR at 12 weeks after protocol start.
